# Integrated Network-Based Analysis of Diseases Associated with Amyloid Deposition Through a Disease–Protein–Drug Network

**DOI:** 10.3390/ph17121736

**Published:** 2024-12-22

**Authors:** Aikaterini E. I. Rizou, Georgia I. Nasi, Avgi E. Apostolakou, Meletios A. Dimopoulos, Efstathios Kastritis, Vassiliki A. Iconomidou

**Affiliations:** 1 Section of Cell Biology and Biophysics, Department of Biology, School of Sciences, National and Kapodistrian University of Athens, Panepistimiopolis, 15701 Athens, Greecegnasi@biol.uoa.gr (G.I.N.); avapo@biol.uoa.gr (A.E.A.); 2 Department of Clinical Therapeutics, School of Medicine, National and Kapodistrian University of Athens, 11528 Athens, Greece; mdimop@med.uoa.gr (M.A.D.); ekastritis@med.uoa.gr (E.K.)

**Keywords:** amyloidoses, computational biology, disease–protein–drug network, network biology, protein-misfolding diseases

## Abstract

**Background:** At present, the complexity that governs the associations between different biological entities is understood better than ever before, owing to high-throughput techniques and systems biology. Networks of interactions are necessary not only for the visualization of these complex relationships but also because their analysis tends to be valuable for the extraction of novel biological knowledge. **Methods:** For this reason, we constructed a disease–protein–drug network, focusing on a category of rare protein-misfolding diseases, known as amyloidoses, and on other pathological conditions also associated with amyloid deposition. Apart from the amyloidogenic proteins that self-assemble into fibrils, we also included other co-deposited proteins found in amyloid deposits. **Results:** In this work, protein–protein, protein–drug, and disease–drug associations were collected to create a heterogenous network. Through disease-based and drug-based analyses, we highlighted commonalities between diseases and proposed an approved drug with prospects of repurposing. **Conclusions:** The identified disease associations and drug candidates are proposed for further study that will potentially help treat diseases associated with amyloid deposition.

## 1. Introduction

The last two decades have seen major biotechnological advances in the field of omic technologies and high-throughput methods. The large amount of data from multiple fields required the use of holistic and interdisciplinary approaches. Systems biology aims to model multi-level systems by integrating genomic, proteomic, transcriptomic, and metabolomic data to uncover novel biological insights and predict the consequences of changes in complex biological systems [1]. It is an undisputable fact that the application of bioinformatics workflows is necessary to manage and interpret this large amount of data. Moreover, network biology supports the study of thousands of interactions between different biological entities, such as genes, proteins, metabolites, and diseases.

Barabási et al. [2] stated that networks of interactions between different biomolecules, inside and outside of the cells and between tissues and organs, are involved in the pathogenesis of diseases. Therefore, disease networks contribute towards the understanding of pathogenic mechanisms and the identification of biomarkers and new drugs [2]. In one of the first studies on disease networks, Kwang-Il Goh et al. [3] constructed a network of disorders and genes, where the associations between the disorders represent at least one common gene of interest. Generally, certain hypotheses correlate the structure and organization of a network to cellular functions and diseases. Firstly, the cellular molecules involved in pathogenesis are usually clustered into the same “neighborhood” of the network. Proteins that play a role in the progression of a certain disease have a greater chance of interacting with each other, and consequently, it is likely that in a neighborhood of an already-known molecule involved in a disease, other such molecules will be discovered. Secondly, diseases that share common biomolecules also have phenotypic commonalities [4].

In a broader perspective, multi-layered networks of diseases, drugs, and drug targets are employed to examine complex pathways affected by drugs, shed light on the mechanisms involved, and, most importantly, pinpoint drugs with prospects of repurposing. Drug repurposing or drug repositioning is a method for detecting new pharmacological actions of approved or failed drugs. A key aspect of repurposed drugs is that they have already gone through many stages of clinical studies, which decreases the possibility of failure due to toxicity or non-specific interactions. As a result, the drug development time and cost, which could reach ten years and 2 billion, respectively, are reduced significantly [5]. A well-known example of drug repurposing is thalidomide, which was originally administered as an anti-emetic during pregnancy. Its tragic side effects led to its removal from the market, but today, it is used to treat leprosy and myeloma due to its anti-inflammatory properties and the ability to inhibit angiogenesis [6].

Our investigation focused on a heterogenous group of protein-misfolding diseases called amyloidoses, characterized by the deposition of misfolded protein aggregates, known as amyloid fibrils, in tissues and organs. These pathological deposits disrupt the normal cellular function, leading to progressive organ damage and potentially death [7]. Under unknown circumstances, soluble proteins lose their native three-dimensional structure, self-assemble into fibrils, and aggregate into insoluble extracellular deposits or intracellular inclusions, causing cell death and organ failure. Notable examples of amyloid-related diseases include Alzheimer’s disease, Parkinson’s disease, systemic amyloidosis, and transthyretin amyloidosis, all of which impose a significant burden on global health. Furthermore, amyloid deposits were observed in a variety of disorders, such as type II diabetes, rheumatoid arthritis, and many types of cancer [8]. Typically, amyloid deposits consist of amyloidogenic and other co-deposited common proteins, like clusterin [9], apolipoprotein E [10], and serum amyloid P-component [11], with a role in amyloidogenesis that has not yet clarified and is still in dispute [12]. This year, the ISA Nomenclature Committee updated its recommendations on amyloid fibril proteins, now totaling 42 in humans [13]. Nevertheless, these co-deposited proteins possibly play a key role in inhibiting the aggregation of the amyloidogenic proteins or protecting the amyloid deposits from degradation [14]. Currently, only a limited number of effective treatments for disorders related to amyloids exist and the majority of them do not directly target the amyloidogenic proteins. The shared pathological hallmark of protein misfolding in amyloidoses makes these disorders particularly intriguing for scientific investigation. This common mechanism provides a foundation for understanding diverse disease manifestations and paves the way for developing strategies that could address multiple conditions within this group [15].

Previous studies of amyloidoses utilizing biological networks were limited to a single disease (e.g., type 2 diabetes [16]) or a group of diseases (e.g., neurodegenerative diseases [17]). In this study, we constructed a specialized disease–protein–drug network for amyloidoses and pathological conditions associated with amyloid deposition, amyloidogenic and co-deposited proteins, and drugs, as well. Finally, this work highlighted common molecules between diseases that could be tested as pharmacological targets and suggested promising approved drugs with the prospects of repurposing.

## 2. Results and Discussion

**Figure 1:**
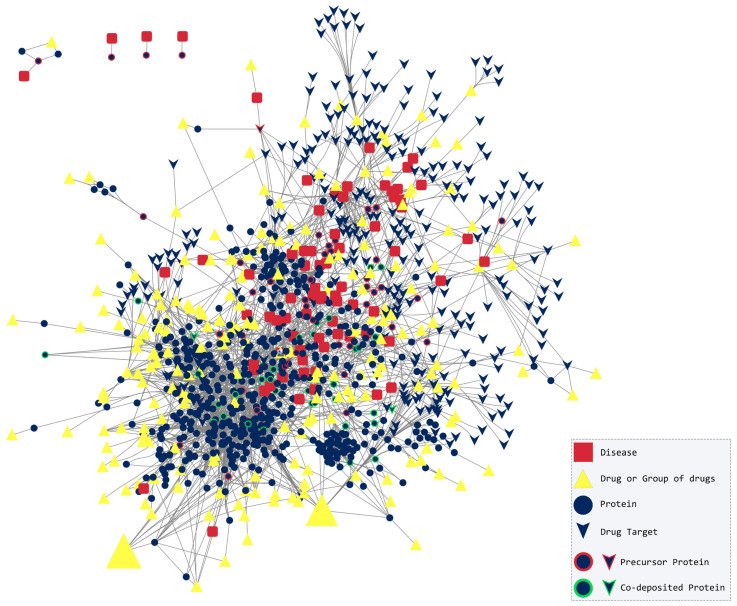
The disease–protein–drug network with 2347 nodes and 7114 edges. Each amyloidosis or pathological condition associated with amyloid deposition (red-colored square) is connected to the amyloidogenic and co-deposited proteins (blue-colored ellipse outlined with red or green, respectively) found in amyloid plaques. Drugs that are indicated for each disease or interact with proteins are represented with a yellow node. A size gradient is used for the number of drugs in each group containing drugs that interact with the same node. Isolated atrial amyloidosis created an isolated network with only three proteins and one drug (upper left corner). Hereditary lysozyme amyloidosis, calcifying epithelial odontogenic tumor and senile seminal vesicle amyloidosis do not have any connection to the rest of the network.

The disease–protein–drug network, as shown in Figure 1, consists of 2347 nodes and 7114 edges. More specifically, among the nodes, there are 76 diseases (amyloidoses or pathological conditions related to amyloid deposition), 854 proteins (amyloidogenic, co-deposited, interactors, and drug targets), and 1414 drugs. In total, 4699 non-redundant protein–protein interactions were added to the final dataset. Four diseases (isolated atrial amyloidosis, hereditary lysozyme amyloidosis, calcifying epithelial odontogenic tumor and senile seminal vesicle amyloidosis) and the amyloidogenic proteins related to them do not share any connections to the rest of the network; hence, they remained unconnected.

### 2.1. Topological Analysis

Although networks of interactions are indisputably useful to visualize the complex relationships between diseases and molecules, the extraction of significant biological information requires the calculation of complex topological parameters. Therefore, the diameter, the density, the average number of neighbors, the heterogeneity, the characteristic path length, and the clustering coefficient of the constructed network, shown in Supplementary Table S1, were calculated. These parameters were compared with those of a random network with the same number of nodes and edges to verify that our network has the properties of a biological network.

First of all, biological networks tend to be sparsely interconnected to ensure that the network connectivity is maintained, even if a node is lost [18]. Indeed, the value of the density was much lower than 0.1 (0.003), meaning that our network was sparse. Additionally, according to the Watts and Strogatz model, biological networks characterized by high clustering coefficient and low characteristic path length have a small-world topology. This topology implies that changes that involve one node may have an impact on the whole network [19]. In our case, the nodes tend to organize in highly connected clusters, as the clustering coefficient of the network was significantly higher (0.158) than that of the random network (0.002). Furthermore, the characteristic path length was approximately five, meaning that we can reach from any node to any other node with five steps (edges). Considering that the clustering coefficient is high, and the characteristic path length is low, the network seems to have small-world properties.

Another noteworthy characteristic of biological networks is that they are scale-free. The degree distribution of the scale-free networks follows the power of law (P(k)~k^−γ^), and there are central nodes that connect to plenty of other nodes and act as hubs. In scale-free networks where the degree exponent is lower than two (γ < 2), hubs play an important role and ensure the integrity of the network after a random node failure [20]. In fact, the degree distribution shown in Supplementary Figure S1 followed the power of law and the equation that describes the relationship between the probability P(k) of a node to interact with k nodes was P(k) = 674.49^−1.416^. Supplementary Table S8 presents the degrees of the nodes in descending order for each category of nodes (diseases, proteins, and drugs). Regarding the diseases that act as hubs, we observed that many infamous pathological conditions are included, for example, rheumatoid arthritis (59 neighbors), type 2 diabetes mellitus (51 neighbors), ankylosing spondylitis (38 neighbors), and Parkinson’s disease (28 neighbors). Because millions of patients suffer from these devastating but well-studied disorders, the majority of their neighbors are drugs, as expected.

### 2.2. The Amyloidogenic Proteins

Generally, the conditions that govern the aggregation of the amyloidogenic proteins to amyloid fibrils are still unknown [21]. In our network, almost half of the amyloidogenic proteins are not well-studied as they are not targeted by any drug and their protein interactors are limited, such as cystatin C (*CST3*), natriuretic peptide A (*NPPA*), and leukocyte cell-derived chemotaxin-2 (*LECT2*). On the other hand, the protein that interacts with the maximum number of drugs (56) is transthyretin, which is found in amyloid deposits in patients suffering from rheumatoid arthritis, wild-type transthyretin-related amyloidosis, apolipoprotein A-I associated amyloidosis, Sjogren’s syndrome, and hereditary transthyretin-related amyloidosis. One unanticipated finding was that serum amyloid A1 and A2, two proteins that self-assemble to fibrils in 21 disorders, shown in Supplementary Figure S2, do not interact with any drug. In addition, no high-confidence protein–protein interactions exist in STRING for these proteins. Consequently, it is apparent that the majority of the amyloidogenic proteins are not studied in detail, and their protein–protein and drug–protein interactions are still to be uncovered.

### 2.3. The Network of Diseases

**Figure 2:**
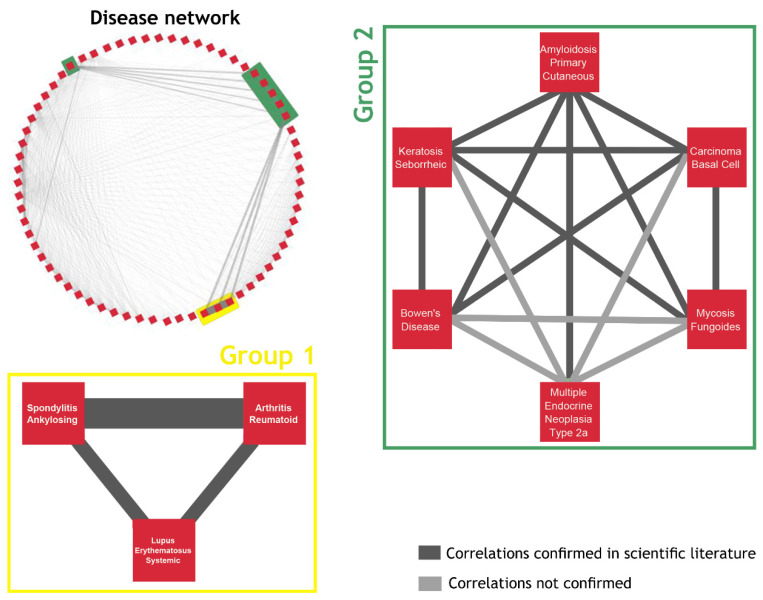
The network of diseases. After the comparison of the subnetworks of the 76 diseases and based on their common neighbors, we constructed the network of diseases. Two diseases are connected if they share at least one neighbor. The width of the edges corresponds to the number of shared neighbors. The diseases in group 1 (yellow box) have the greatest number of neighbors in common, while the six diseases in group 2 (green box) are all connected to each other. The majority of the correlations between each pair of diseases were confirmed by the scientific literature (dark grey line). The light grey lines represent the correlations that need to be further investigated.

Because of the size and the complexity of the disease–protein–drug network, we used two different approaches, starting from diseases and from drugs (next section), to extract new biological information. In the first approach, we created a network that only consisted of diseases, and its purpose was to highlight commonalities between them. In total, the 76 disease subnetworks were compared to each other, and the number of common first neighbors between each pair of diseases was used as a weight to create a weighted disease network (Supplementary Table S7). It is worth mentioning that two groups stood out, a “triangle” of three diseases (Figure 2, Group 1) and a densely connected group of six diseases (Figure 2, Group 2).

**Figure 3:**
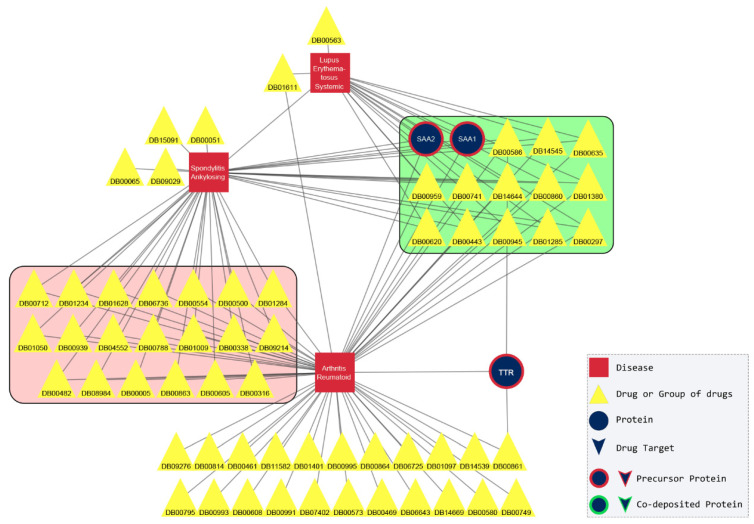
A merged network of systemic lupus erythematosus, spondylitis ankylosing, and rheumatoid arthritis subnetworks. The three diseases seem to be correlated to each other and formed a densely connected triangle that stood out from the network of diseases. Their correlation was confirmed in scientific literature. The common drugs and proteins of the three diseases are shown in the green box. The drugs in the pink box correspond to those shared between ankylosing spondylitis and rheumatoid arthritis. The proteins are labeled with their corresponding gene name and the drugs with DrugBank ID (Supplementary Materials).

The merged subnetworks of Group 1 are depicted in Figure 3, where rheumatoid arthritis (RA) shares 34 drugs and proteins with spondylitis ankylosing (SA) and 16 with systemic lupus erythematosus (SLE). SA and SLE connect to each other through 14 common nodes. Concerning the common neighbors between these three diseases, we found 12 drugs, most of which are characterized as anti-inflammatory or anesthetic, and two amyloidogenic proteins, serum amyloid A1 and A2. To confirm these predicted associations between the disorders, we performed an extensive literature search and indeed, there are correlations between the three diseases. Akhondi and Varacallo supported the idea that RA and SA have many symptoms in common, even though the genetic background is different [22]. In the same way, RA shares many mechanisms of the immune system with SLE, but the pathology and the treatment are different [23,24]. The correlation between SA and SLE is still under investigation [25,26]. Regarding the serum amyloid A1 and A2 proteins, several studies suggested that their levels in serum were indicative of the progression in all three diseases [27,28,29].

**Figure 4:**
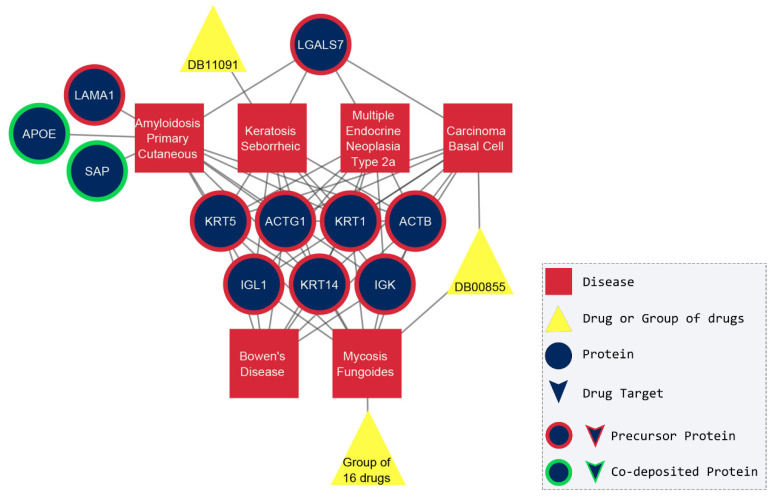
The common neighbors between the group of six diseases. Bowen’s disease, basal cell carcinoma, mycosis fungoides, multiple endocrine neoplasia type 2a, primary cutaneous amyloidosis, and seborrheic keratosis share seven amyloidogenic proteins and one drug. The correlation between them was confirmed in scientific literature. The only disease that has no co-references with any other in the literature is multiple endocrine neoplasia type 2a and needs further investigation. The proteins are labeled with their corresponding gene name and the drugs with DrugBank ID (Supplementary Materials).

The second group of densely connected diseases that emerged included basal cell carcinoma, seborrheic keratosis, mycosis fungoides, multiple endocrine neoplasia type 2a, primary cutaneous amyloidosis, and Bowen’s disease. The main observation was that seven amyloidogenic proteins are the common nodes that connect all six diseases, as depicted in Figure 4. Even though a considerable number of publications clinically investigate the commonalities of these diseases, a systematic understanding of a molecular basis is still lacking. Nevertheless, the existing research suggests that some of these diseases mimic each other, like Bowen’s disease and seborrheic keratosis [30], while the distinction between them becomes difficult, for instance, seborrheic keratosis with basal cell carcinoma or mycosis fungoides [31,32,33,34,35]. In addition, the same treatment had a positive outcome in the case of basal cell carcinoma and mycosis fungoides [36], and Bowen’s disease and basal cell carcinoma [37,38]. Surprisingly, it was already established that cutaneous amyloidosis can emerge secondarily and co-exist with all these four skin disorders [39]. The mechanism of the amyloidogenesis in apoptotic keratinocytes and the etiology behind cutaneous amyloidosis is not fully understood, although immunohistochemical studies have proven that the major components of cutaneous amyloid deposits are keratins (type I cytoskeletal 14—*KRT14*, type II cytoskeletal 1—*KRT1*, type II cytoskeletal 5—*KRT5*), actin (actin cytoplasmic 1—*ACTB* and 2—*ACTG1*), and galectin-7 (*LGALS7*) [40,41,42,43]. Last but not least, the sixth disease in this group, multiple endocrine neoplasia type 2a, is also associated with the most common type of cutaneous amyloidosis, the cutaneous lichen amyloidosis [42,44], and patients suffering from both have been documented [45]. In spite of the fact that multiple endocrine neoplasia type 2a have the same number of common proteins as the rest of the group, there are no scientific publications investigating their possible connection yet. In conclusion, most of our results are in accordance with the existing literature (Figure 4), as there is a growing number of studies stating that the six diseases are indeed correlated.

### 2.4. Drug-Based Approach

The purpose of this last section was to discover drugs with possible alternative uses, and for this reason, we present a case study of a drug with prospects of repurposing. Having extracted the 76 disease subnetworks, we carried out extensive literature research in PubMed and DrugBank and focused only on the drugs that target the amyloidogenic proteins but have no reference with the corresponding disease of the subnetwork. Another criterion taken under consideration was the topological parameters of these drugs in the disease–protein–drug network. More specifically, prior research groups have already noted the importance of betweenness centrality in drug repurposing [46]. Betweenness centrality is a measure of the shortest paths passing through a node that acts like a bridge connecting different parts of a network [47]. Therefore, a drug with a high betweenness centrality score is more likely to be administrated for several therapeutic uses and, consequently, is a good candidate for repurposing [48]. The drug that fulfilled all three criteria was Polaprezinc (DB09221), which targets the superoxide dismutase [Cu-Zn] (*SOD1*), an amyloidogenic protein involved in amyotrophic lateral sclerosis. Polaprezinc, a chelated form of l-carnosine and zinc, is indicated for gastric ulcers [49]. In vivo studies showed that it increased the concentration of the antioxidant enzyme dismutase, which takes part in the conversion of superoxide to molecular oxygen and hydrogen peroxide. The pharmacological action is similar to that of Selegiline (DB01037), a monoamine oxidase inhibitor that reduces oxidative stress and is used to treat Parkinson’s [50].

**Figure 5:**
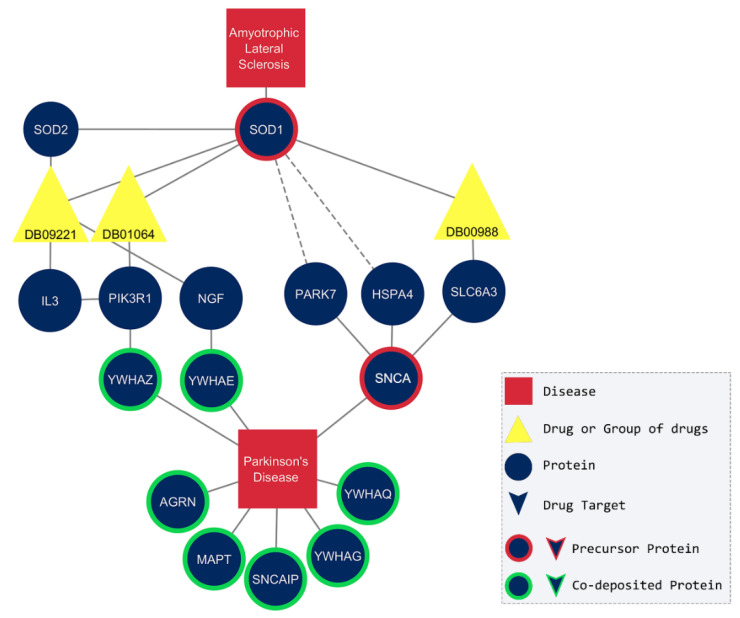
Parkinson’s disease and amyotrophic lateral sclerosis share first and second neighbors. The dotted lines represent the medium-confidence (0.700) interactions according to STRING database. Polaprezinc (DB09221) is proposed to be an ideal candidate for the treatment of both diseases. The proteins are labeled with their corresponding gene name and the drugs with DrugBank ID (Supplementary Materials).

In Figure 5, it is obvious that the neighbors of the superoxide dismutase interact with proteins that participate in the subnetwork of Parkinson’s disease. For example, Isoprenaline (DB01064) and Dopamine (DB00988) interact with interleukin-3 (*IL3*), phosphatidylinositol 3-kinase regulatory subunit alpha (*PIK3R1*), beta-nerve growth factor (*NGF*), and sodium-dependent dopamine transporter (*SLC6A3*). Moreover, the superoxide dismutase interacts with medium confidence (0.700) with the Parkinson disease protein 7 (*PARK7*) and heat shock protein 7 (*HSP7*), two close neighbors of α-synuclein (*SNCA*). In fact, the review of Eleutherio et al. [51] provided well-known information about the role of superoxide dismutase in amyotrophic lateral sclerosis and Parkinson’s disease and suggested that a common treatment should be investigated.

Considering our results, we proposed that Polaprezinc should be tested for treatment in amyotrophic lateral sclerosis and Parkinson’s disease in combination with stabilizing compounds. For example, one of the most promising compounds is diacetyl-bis (4-ethylthiosemicarbazonato) copperII (Cu^II^[atsm]), which is currently tested for both diseases (ClinicalTrials.gov unique ID NCT02870634) [52]. In addition, the drug was not tested for these two diseases before as there are no scientific publications with the terms “polaprezinc” AND “amyotrophic lateral sclerosis”, or “polaprezinc” AND “Parkinson’s disease”, or similar terms (last search on February 2023). Last but not least, the neuroprotective functions of the main component of the drug, l-carnosine, were already established [53], but its role in amyotrophic lateral sclerosis is still unknown.

## 3. Materials and Methods

**Figure 6:**
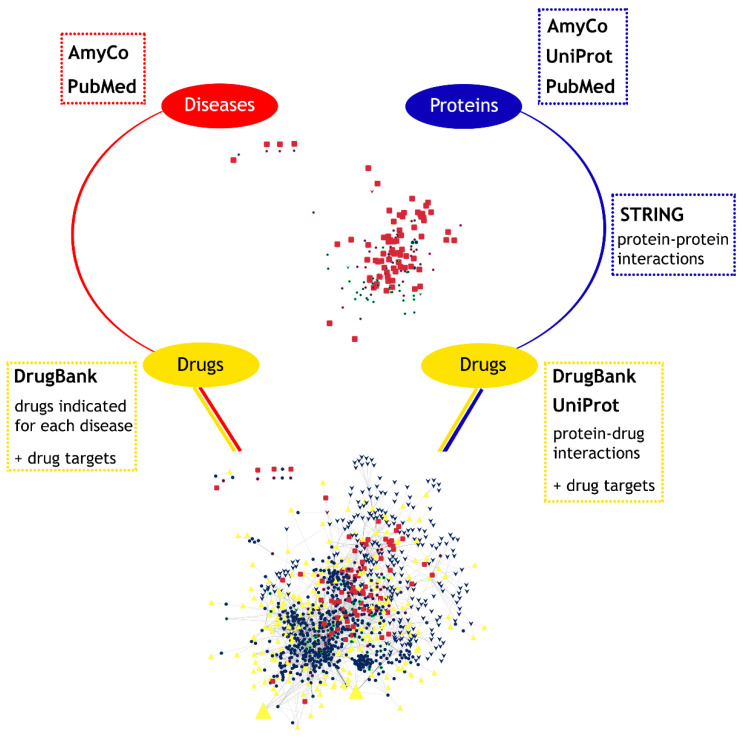
The study design for the creation of the disease–protein–drug network. The 76 diseases associated with amyloid deposition and the proteins involved were collected from the AmyCo database and the scientific literature. Protein–protein interactions were gathered from STRING, while protein–drug interactions were gathered from DrugBank and UniProt. Finally, we added the drugs indicated for each disease and their drug targets from DrugBank.

To gain a broader perspective on the available treatments in diseases associated with amyloid deposition, we constructed a heterogenous and complex disease–protein–drug network. Four different datasets were collected: the associations between diseases and the proteins found on amyloid deposits, the protein–protein interactions, the protein–drug interactions, and the drugs indicated for each disease. A brief description of the methodology for the construction of the network is presented in Figure 6.

### 3.1. Disease–Protein Associations

AmyCo is a comprehensive resource that collects and classifies amyloidoses and pathological conditions related to the deposition of amyloid fibrils [54]. The database contains 49 amyloidoses and 36 other disorders, collected after a manual literature search. Each unique entry provided information about the disease, such as the name according to the International Association of Amyloidosis (ISA), the ICD-10 categorization, the alternative names, and the affected tissue. Moreover, the amyloidogenic and the co-deposited proteins found in each disease were characterized by the unique UniProt accession number (AC), the gene name, the alternative names, the sequence, and the length. The database was downloaded as an XML file, and the connections between the 75 diseases and the associated proteins were extracted.

In an attempt to check for conditions newly characterized as amyloidoses, we performed an extensive search in PubMed (May 2022), and one more disease was added to the dataset, somatostatin-related amyloidosis [55]. This brings the final total of diseases to 76 (Supplementary Table S2).

### 3.2. Protein–Protein Interactions

Protein–protein interactions were gathered from the freely available database STRING, which integrates experimentally validated and predicted connections between proteins, including physical and functional interactions. STRING offers curated and comprehensive data while combining various sources, including experimental data, computational predictions, and text mining [56]. A combined score from zero to one estimates the confidence of the interaction from high confidence (<0.900) to low confidence (<0.150). Since many amyloidogenic proteins are still understudied, we chose this proteomic database to collect as many high-confidence interactions as possible. For this reason, we selected only the physical interactions of the amyloidogenic and the co-deposited proteins through STRING API (version 11.5) with a minimum required interaction score of 0.900 (Supplementary Table S3). The number of neighbors per protein was limited to 100. Finally, all non-human interactions were filtered out.

### 3.3. Drug–Disease and Drug-Protein Interactions

DrugBank 5.0 [49] is an informative database that contains not only approved drugs but also experimental drugs, as well as information about their structure, pharmacokinetic properties, uses, interactions with biological molecules, and pharmacological targets. In this case, we performed a search to export the drugs indicated for each disease and their interactions with proteins (May 2022) (Supplementary Table S4). In addition, for the AmyCo proteins and their interactors, we retrieved their drug interactions from DrugBank cross-reference in UniProt (query field: database: (type: drugbank)) (Supplementary Table S5). UniProt contains well-annotated records and offers full programmatic access. Finally, the dataset does not include zinc, copper, or aluminum, as they interact non-specifically with many proteins.

### 3.4. Disease–Protein–Drug Interactions Network

We collected the final dataset of associations between the following interactions:

(1) Diseases and proteins;
(2) Proteins and proteins;
(3) Proteins and drugs;
(4) Diseases and drugs.

We then proceeded to the creation of the network and the calculation of its topological features. For the visualization and analysis of such complex graphs, we used the freely available platform Cytoscape 3.9.1 [57]. This open-source tool provides plenty of apps to import data from various databases, perform clustering and enrichment analysis, etc. From these apps, we selected NetworkAnalyzer [58] and Network Randomizer [59] to better understand the properties of our network. The first one is a pre-installed app that calculates many topological parameters, such as node degree, diameter and radius, average clustering coefficient, topological coefficient, and heterogeneity. The second one creates random networks of the same number of nodes and edges with the given network, which can be analyzed to compare their topological features to those of our network. This comparison could demonstrate that the network is not random and, in combination with the topological analysis, shows that it, instead, has the properties of a biological network. The degree distribution chart was constructed through RStudio [60] using the statistical programming language R and the packages “igraph”, “powerLaw”, and “ggplot2”.

### 3.5. A Disease-Based and Drug-Based Approach

Due to the heterogeneity, complexity, and size of our network, we applied two different approaches, one starting from diseases and another starting from drugs, in an attempt to extract valuable biological information.

In the first approach, we constructed a second network that depicts the commonalities between the diseases. For this reason, the first neighbors of each disease were isolated, and those 76 subnetworks were all compared to each other. As a result, a 2D matrix with the number of common nodes between every pair of diseases was constructed. These numbers were used as weights in a network that connects diseases with at least one common neighbor (protein or drug) (Supplementary Table S7). The edges with zero weight were excluded from this network. The two groups of diseases that stood out from the network of diseases are discussed below. The importance of these associations was verified by reviewing the scientific literature.

In the second approach, we started our analysis from the drugs targeting the amyloidogenic proteins. The goal was to select those drugs that did not have any co-reference with the corresponding disease of the subnetwork in the scientific literature. Then, we chose the drugs with the highest betweenness centrality, and their subnetworks were studied in detail in order to find other diseases or amyloidogenic proteins close to that drug.

## 4. Conclusions

In this study, we employed a network-based approach that has been widely used in previous research to examine the interactions between diseases associated with amyloid deposition, drugs, and proteins. Previous approaches were limited in scope, typically to one or few diseases and, therefore, could not provide insights into the interconnectedness of these diseases. This methodology allowed us to explore the complex relationships among these entities, revealing insights into disease mechanisms and potential therapeutic strategies. By analyzing these networks, we can identify key proteins involved in disease progression and evaluate how existing drugs may interact with these proteins, contributing to the development of novel treatment options or drug repurposing opportunities. This is the first work of this scope and provides a comprehensive assessment of the drugs associated with a category of rare protein misfolding diseases, known as amyloidoses, and disorders associated with amyloid deposition. The initial objective of this work was to create a disease–protein–drug network and through disease-based and drug-based analyses, we tried to discover common neighbors between these pathological conditions and propose novel uses for existing drugs. Since some of these diseases are well-researched, such as Alzheimer’s disease or Parkinson’s disease, compared to other rarer disorders like Meretoja syndrome or limb–girdle muscular dystrophy, type 2B, it is, therefore, mandatory to experimentally validate our in silico hypothesis. Further in vitro and in vivo studies could validate the proposed associations between the densely connected groups of diseases and test the promising approved drug against amyotrophic lateral sclerosis and Parkinson’s disease.

## Data Availability

The original contributions presented in this study are included in the article/Supplementary Materials. Further inquiries can be directed to the corresponding author.

## Supplementary Materials

The following supporting information can be downloaded at: https://www.mdpi.com/article/10.3390/ph17121736/s1. Table S1: describes the topological parameters of the constructed network compared to those of a random network; Table S2: contains the diseases (amyloidosis and disorders related to amyloid deposition) and the amyloidogenic or co-deposited proteins extracted from AmyCo database or scientific literature; Table S3: contains all high-confidence (0.900) protein-protein interactions collected from STRING database; Table S4: collects all drug-protein interactions that were extracted from DrugBank and UniProt; Table S5: has the drugs indicated for each disease collected from DrugBank; Table S6: contains the categorization of each node to disease, drug, protein, drug target, precursor, or co-deposited protein; Table S7: collects all the predicted associations between the 76 diseases. The weight of each edge corresponds to the number of common neighbors. The edges with zero weight were excluded; Table S8: contains the degree of each node categorized in diseases, proteins, and drugs; Table S9: contains the betweenness centrality of each node categorized in diseases, proteins, and drugs; Figure S1: The node degree distribution emphasizes the network’s scale-free characteristics. The green line shows that the P(k) decays as the power law (P(k) = 674.49–1.416). The axes are in logarithmic scale; Figure S2: The subnetwork of the serum amyloid A1 (*SAA1*) and A2 (*SAA2*) and the 21 diseases that are connected to. The two proteins do not interact with any protein or drug.

## Abbreviations

RA Rheumatoid arthritis; SA Spondylitis ankylosing; SLE Systemic lupus erythematosus.
