# Supplementary material for: Integrated Network-Based Analysis of Diseases Associated with Amyloid Deposition Through a Disease–Protein–Drug Network
Source: Pharmaceuticals (Basel). 2024 Dec 22;17(12):1736. doi: 10.3390/ph17121736 (PMC11677318; doi:10.3390/ph17121736)
Supplement: Supplementary file 1 [file pharmaceuticals-17-01736-s001.zip › Supplementary material.pdf]

# Supplementary material

## Integrated network-based analysis of diseases associated with amyloid deposition through a disease-protein-drug network

Aikaterini E. I. Rizou[a], Georgia I. Nasi[a], Avgi E. Apostolakou[a], Meletios A. Dimopoulos[b], Efstathios Kastritis[b] and Vassiliki A. Iconomidou[a,*]

[a] *Section of Cell Biology and Biophysics, Department of Biology, School of Sciences, National and Kapodistrian University of Athens, Panepistimiopolis, Athens, Greece;* [b]*Department of Clinical Therapeutics, School of Medicine, National and Kapodistrian University of Athens, Athens, Greece*

The drugs are characterized by the unique DrugBank ID and the proteins by the unique UniProt ID.

## Abbreviations

RA rheumatoid arthritis

SA spondylitis ankylosing

SLE systemic lupus erythematosus

## Tables in Data (excel file)

Table S1 describes the topological parameters of the constructed network compared to those of a random network.

Table S2 contains the diseases (amyloidosis and disorders related to amyloid deposition) and the amyloidogenic or co-deposited proteins extracted from AmyCo database or scientific literature.

Table S3 contains all high-confidence (0.900) protein-protein interactions collected from STRING database.

Table S4 collects all drug-protein interactions that were extracted from DrugBank and UniProt.

Table S5 has the drugs indicated for each disease collected from DrugBank.

Table S6 contains the categorization of each node to disease, drug, protein, drug target, precursor, or co-deposited protein.

Table S7 collects all the predicted associations between the 76 diseases. The weight of each edge corresponds to the number of common neighbors. The edges with zero weight were excluded.

Table S8 contains the degree of each node categorized in diseases, proteins, and drugs.

Table S9 contains the betweenness centrality of each node categorized in diseases, proteins, and drugs.

## The genes referred in figures and the corresponding protein.

ACTB actin cytoplasmic 1

ACTG1 actin cytoplasmic 2

AGRN agrinin

APCS P component of serum amyloid

APOE apolipoprotein E

CDH5 cadherin-5

CRBN cereblon protein

CST3 cystatin c

FN1 fibronectin

HSP7 heat shock protein 7

HSPG2 basement membrane-specific heparan sulfate proteoglycan core protein

IGK immunoglobulin kappa light chain

IGL1 immunoglobulin lambda-1 light chain

IL3 interleukin-3

KRT1 keratin type I cytoskeletal 1

KRT14 keratin type I cytoskeletal 14

KRT5 keratin type I cytoskeletal 5

LAMA1 laminin subunit alpha-1

LECT2 leukocyte cell-derived chemotaxin-2

LGALS7 galectin-7

MAPT microtubule associated protein tau

NGF beta-nerve growth factor

NPPA natriuretic peptide A

PARK7 parkinson disease protein 7

PIK3R1 phosphatidylinositol 3-kinase regulatory subunit alpha

PTGS2 prostaglandin G/H synthase 2

SAA1 serum amyloid A1

SAA2 serum amyloid A2

SLC6A3 sodium-dependent dopamine transporter

SNCA a-synuclein

SNCAIP Synphilin-1

SOD1 superoxide dismutase [Cu-Zn]

SOD2 Superoxide Dismutase 2

TNFSF11 tumor necrosis factor ligand superfamily member 11

TTR transthyretin

VEGFA vascular endothelial growth factor-A

YWHAE 14-3-3 protein epsilon

YWHAG 14-3-3 protein gamma

YWHAQ 14-3-3 protein theta

YWHAZ 14-3-3 protein zeta/delta

## The drugs referred in the figures.

DB00005 Etanercept

DB00051 Adalimumab

DB00065 Infliximab

DB00297 Bupivacaine

DB00316 Acetaminophen

DB00338 Omeprazole

DB00443 Betamethasone

DB00461 Nabumetone

DB00469 Tenoxicam

DB00480 Lenalidomide

DB00482 Celecoxib

DB00500 Tolmetin

DB00554 Piroxicam

DB00563 Methotrexate

DB00573 Fenoprofen

DB00580 Valdecoxib

DB00586 Diclofenac

DB00605 Sulindac

DB00608 Chloroquine

DB00620 Triamcinolone

DB00635 Prednisone

DB00712 Flurbiprofen

DB00741 Hydrocortisone

DB00749 Etodolac

DB00788 Naproxen

DB00795 Sulfasalazine

DB00814 Meloxicam

DB00855 Aminolevulinic acid

DB00860 Prednisolone

DB00861 Diflunisal

DB00863 Ranitidine

DB00864 Tacrolimus

DB00939 Meclofenamic acid

DB00945 Aspirin

DB00959 Methylprednisolone

DB00988 Dopamine

DB00991 Oxaprozin

DB00993 Azathioprine

DB00995 Auranofin

DB01009 Ketoprofen

DB01037 Selegiline

DB01050 Ibuprofen

DB01064 Isoprenaline

DB01097 Leflunomide

DB01234 Dexamethasone

DB01284 Tetracosactide

DB01285 Corticotropin

DB01380 Cortisone acetate

DB01401 Choline magnesium trisalicylate

DB01611 Hydroxychloroquine

DB01628 Etoricoxib

DB03088 pidolic acid

DB04552 Niflumic acid

DB06643 Denosumab

DB06725 Lornoxicam

DB06736 Aceclofenac

DB07402 Azapropazone

DB07909 (1S,2S,5S)2-(4-GLUTARIDYLBENZYL)-5-PHENYL-1- CYCLOHEXANOL

DB08984 Etofenamate

DB09029 Secukinumab

DB09214 Dexketoprofen

DB09221 Polaprezinc

DB09276 Sodium aurothiomalate

DB09331 Daratumumab

DB11091 Hydrogen peroxide

DB11582 Thiocolchicoside

DB14539 Hydrocortisone acetate

DB14545 Hydrocortisone succinate

DB14644 Methylprednisolone hemisuccinate

DB14669 Betamethasone phosphate

DB15091 Upadacitinib

**Supplementary figures**

**Supplementary figures**

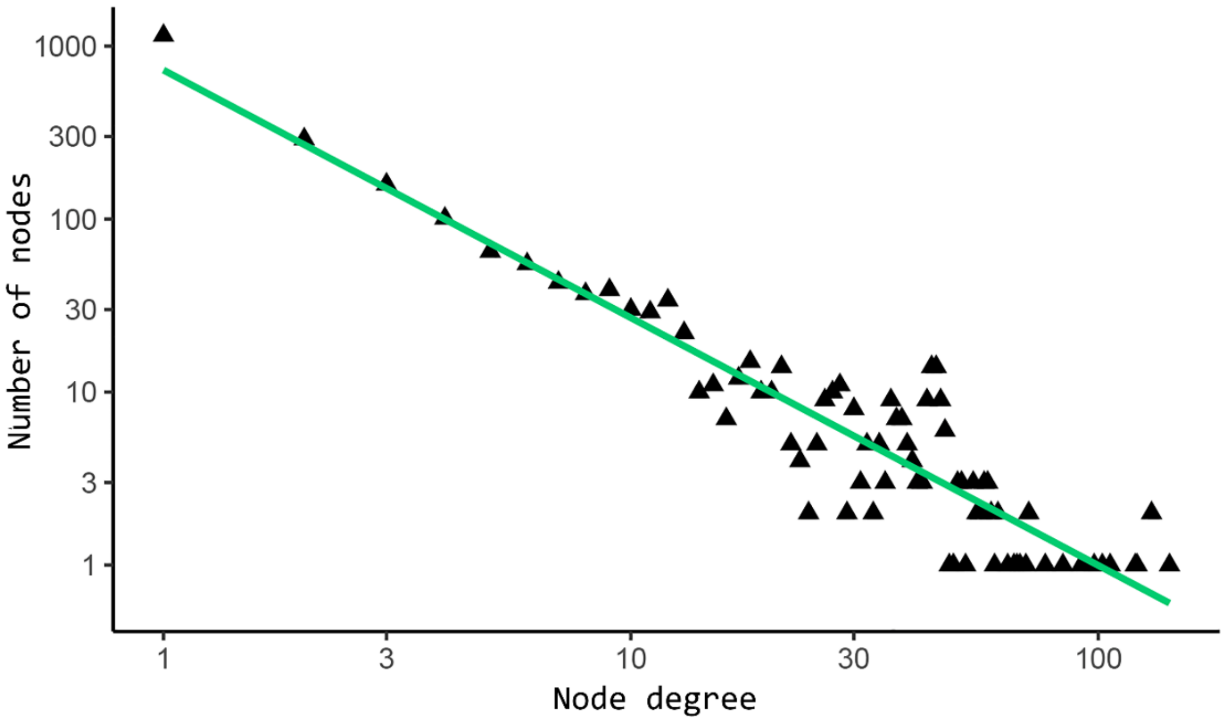


Figure S1. The node degree distribution emphasizes the network's scale-free characteristics. The green line shows that the P(k) decays as the power law (P(k)=674.49-1.416). The axes are in logarithmic scale.

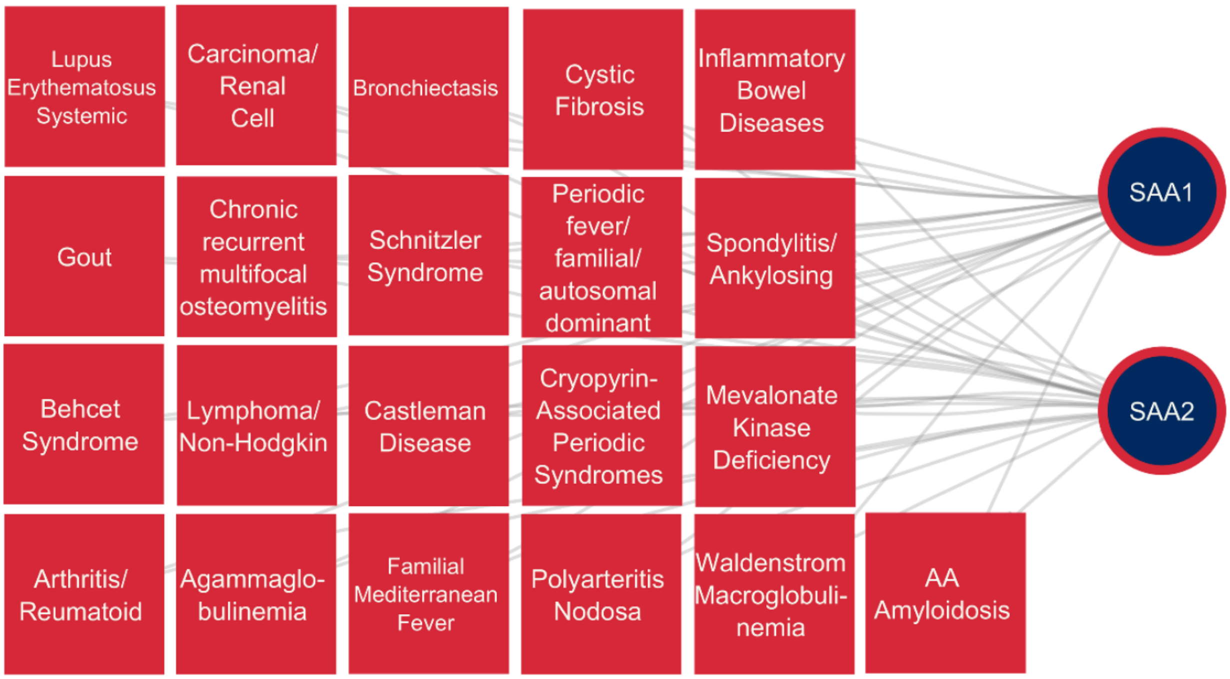

Figure S2. The subnetwork of the serum amyloid A1 (*SAA1*) and A2 (*SAA2*) and the 21 diseases that are connected to. The two proteins do not interact with any protein or drug.
